# Diabetes Health, Residence & Metabolism in Asians: the DHRMA study, research into foods from the Indian subcontinent - a blinded, randomised, placebo controlled trial

**DOI:** 10.1186/1471-2261-11-70

**Published:** 2011-12-02

**Authors:** Jeetesh V Patel, Elizabeth A Hughes, Gregory YH Lip, Paramjit S Gill

**Affiliations:** 1 Metabolic Medicine, Sandwell General Hospital, Sandwell and West Birmingham Hospitals NHS Trust, Lyndon, West Bromwich, Lyndon (B71 4HJ), UK; 2 University of Birmingham Centre for Cardiovascular Sciences, City Hospital, Sandwell and West Birmingham Hospitals NHS Trust, Dudley Rd, Birmingham (B18 7QH) UK; 3 Primary Care Clinical Sciences, University of Birmingham, Vincent Drive, Birmingham, (B15 2TT) UK

## Abstract

**Background:**

Coronary heart disease (CHD) is highly prevalent amongst the South Asian communities in Britain. The reasons for this excess CHD risk are multifactorial, but in part relate to a susceptibility to diabetes mellitus - where the aberrant metabolism of non-esterified fatty acids (NEFA) and glucose are likely to underpin vascular disease in this population. Dietary intervention is an important and first line approach to manage increased CHD risk. However, there is limited information on the impact of the South Asian diet on CHD risk.

**Methods/Design:**

The Diabetes Health, Residence & Metabolism in Asians (DHRMA) study is a blinded, randomised, placebo controlled trial that analyses the efficacy of reduced glycaemic index (GI) staples of the South Asian diet, in relation to cardio-metabolic risk factors that are commonly perturbed amongst South Asian populations - primarily glucose, fatty acid and lipoprotein metabolism and central adiposity. Using a 10-week dietary intervention study, 50 healthy South Asians will be randomised to receive either a DHRMA (reduced GI) supply of chapatti (bread), stone ground, high protein wheat flour and white basmati rice (high bran, unpolished) or commercially available (leading brand) versions chapatti wheat flour and basmati rice. Volunteers will be asked to complete a 75g oral glucose tolerance test at baseline and at 10-weeks follow-up, where blood metabolites and hormones, blood pressure and anthropometry will also be assessed in a standardised manner.

**Discussion:**

It is anticipated that the information collected from this study help develop healthy diet options specific (but not exclusive) for South Asian ethnic communities.

Trial registration

Current Controlled Trials ISRCTN02839188

## Background

Coronary heart disease (CHD) is the leading cause of mortality and morbidity amongst South Asians living in the UK (people originating from the Indian subcontinent), who have a 50% greater risk of dying prematurely from this affliction than the general population [1,2]. This increased CHD risk is multifactorial, but in part is likely to be influenced by diabetes mellitus [3], which is more common amongst South Asian groups [4]. Indeed, CHD risk in South Asians is coordinated with the frequent clustering of metabolic risk factors that includes aberrant glucose and lipid metabolism[5,6], which may represent the commonality between diabetogenesis and atherogenesis in this population. These metabolic risk factors are recognised to be associated with more advanced atherosclerotic vascular disease [7] and increased cardiovascular mortality [8,9].

There is an urgent need for effective 'targeted' approaches in the prevention and management of CHD and the associated metabolic risk in South Asians. Dietary intervention remains a 'first line' approach to manage this increased risk. While dietary health promotion approaches to the management and prevention of CHD is availible for South Asians (i.e diet leaflets and health DVDs, websites such as South Asian health [10]), much of the scientific evidence that such material is based upon uses findings in populations other than South Asians. For South Asian diabetics, general health promotion material has had limited impact [11]. South Asians have also been excluded from major diabetes and cardiovascular clinical trials [12]. Reasons for this are complex, and it is not clear what the range of contributory factors are, what are the main reasons and whether the real issue is one of 'planned exclusion', 'inadvertent exclusion', 'non-participation' or a mixture of these. There is a need to increase South Asian participation in research.

### Dietary transition and cardiovascular risk in South Asians

The present study [DHRMA] was specifically developed to advance findings from an epidemiological study of CHD risk in a population of Indian migrants living in the UK. In this study, nutritional intake, lifestyle, glucose tolerance and CHD risk were compared between Indians living in Sandwell (UK) and their contemporaries still living in villages of origin in rural India [13]. While diabetes prevalence was generally high (15% irrespective of site), in India it was less frequently accompanied by metabolic CHD risk factors such as raised triglycerides and central obesity. The greater metabolic-CHD risk amongst the migrant Indians was underpinned by dietary change, a transition towards higher energy and fat intake at the expense of carbohydrate. Data also suggested a background of higher glucose excursions and unregulated NEFA metabolism in Indian populations [14], which has been reported elsewhere as ethnic differences in NEFA profiles following oral glucose challenges [15]. NEFA metabolism may be a key driver for the major CHD risk of these communities, through its role on lipid metabolism and hepatic insulin resistance [16].

### Glycaemic index in South Asian foods

Reducing postprandial glucose and NEFA concentrations may be a desirable option in the dietary management of metabolic-CHD risk in South Asians [17]. In recent years the 'glycaemic index' (GI) (a ranking system for carbohydrates based on their immediate effect on blood glucose levels) has emerged as a classification system for carbohydrate-containing food, where consumption of high GI meals in comparison with energy- and nutrient-controlled low GI foods are adversely associated with glucose intolerance [18]. The GI of foods relates to the nature of monosaccharides, starches, fat, protein and fibre it contains, and the method with which it is cooked. Typically, stone-ground breads have a low GI (high protein and fibre), which is why stone-ground chapatti flour is also likely to have a low GI, compared to other more refined versions (devoid of bran). Basmati rice has a high amylase content, which confers a low GI, and a higher bran content is likely to lower GI further, especially in an unpolished (i.e. high bran) preparation. Importantly, low GI foods are shown to reduce hyperglycaemia, hyperinsulinaemia and postprandial circulating NEFA [19]. While South Asians resident in Britain have varied diets (influenced by culture, religion and British culture), many migrants still subscribe to diets that include staples of carbohydrate such as breads (chapatti, roti, naan) and rice [20]. The industrialisation of food processing and relative affluence has promoted the consumption of more refined foods (high GI) both in India and the UK. In South India [21], postprandial glucose excursions were favourably delayed by staple foods prepared and eaten there, but these foods have yet to be assessed in Britain amongst Indian migrant populations.

### Objectives and hypothesis of DHRMA

The purpose of DHRMA is to provide the scientific basis to help regenerate cultural traditions in food intake, with the aim to develop dietary CHD management options, specifically tailored (but not exclusively) for people of South Asian origin living in the UK. DHRMA is a novel project, as these foods are not commercially available, and the study plan is based on pilot findings from our own research and developed with experience from our ongoing programme of CHD risk screening in ethnic minorities (Healthy Hearts), where 900 individuals have been assessed using a community based approach. [22]

### Objectives are as follows

(i) to establish the GI of existing (ie. produced in the UK) traditional, and regenerated (see (ii)) food staples of South Asian cuisine (specifically chapatti flour and basmati rice).

(ii) to develop will DHRMA test foods that have been specifically that are processed in a similar fashion to those available in rural India: basmati rice that is polished to a low level (less bran removed, grain quality distribution ratio score by KETT (Tokyo, Japan) of <35%)*, and stone-ground chapatti flour with added grains (oats, barley and chick pea).

(iii)to determine the efficacy of low GI DHRMA foods on indices of diabetes and CHD risk using a blinded, randomised controlled trial.

Note* KETT is an industry standard originating from Japan - a score based on the whiteness of rice. Generally amongst basmati rice sold in the UK, this score is around 40-44%.

The DHRMA study will focus on testing the hypothesis that a 10-week supply of DHRMA foods (reduced GI supply of chapatti stone ground, high protein wheat flour and high bran, unpolished white basmati rice) to healthy South Asians will result in favourable changes in cardio-metabolic risk factors (glucose tolerance, fatty acid, triglyceride and HDL cholesterol metabolism and central adiposity) as compared those randomised to commercially popular (market leaders) versions of chapatti flour and white basmati rice.

## Methods/Design

Ethical approval for the study was provided by North Staffordshire Research Ethics Committee (REC reference 08/H1204/130), and all prospective volunteers for the DHRMA study will undergo an informed consent process. The DHRMA study is split into two parts, (i) the assessment of glycaemic index in Indian foods and (ii) a dietary intervention trial.

### Development of DHRMA low glycaemic index foods

The Study will be undertaken in the area of Sandwell and West Birmingham, at Sandwell and West Birmingham Hospitals NHS trust (UK). Healthy volunteers (body mass-index between 18-25 kg/m^2^, no known cardiovascular disease or associated medication, normal glucose tolerance) will be approached for this study from local communities [22] as well as hospital staff. The GI will be measured using FAO/WHO expert consultation (1998) guidelines. Briefly, an eight point capillary blood glucose profile will be measured over a 2 hour period (i.e. 15 min intervals) following the administration of a 50 g glucose load in each prospective volunteer after an overnight fast. Volunteers will then be invited for repeat visits where a 50 g glucose equivalent from test foods will be administered (done in triplicate for as recommended by the FAO/WHO guidelines).

The preparation of chapattis (ingredients, size and cooking time) and rice (water added and cooking time) will be standardised by researchers and subjects will be advised a standard level of activity during the study period. The test foods will be eaten over 15 minutes with water. Capillary blood samples will be analysed using a standard capillary glucose monitor (quality assurance monitored, Roche Accutrend GC, Roche UK). A capillary blood glucose response for curve for each test food will be plotted, and with each subject, the GI for the food will be the proportion of this area as a percentage of the response to 50 g of glucose.

### Interventional trial

This is a 10-week, blinded, parallel, randomised intervention trial in South Asians either DHRMA versions of stone ground chapatti flour and high bran white Basmati rice or the commercially popular white chapatti flour (Elephant atta, Premier Foods, UK) and white Basmati (Tilda, UK) will be supplied to participants.

The Inclusion criteria will include age <55 yrs, BMI <30 kg/m^2^, systolic/diastolic BP <140/90 mmHg (no antihypertensive use), South Asian ethnicity and use of chapatti flour or rice as part of their normal diet (>3 times a week) and >50% of energy derived from carbohydrates and <30% from fat - determined by 4-day food diaries, used previously [13]).

**Figure 1:**
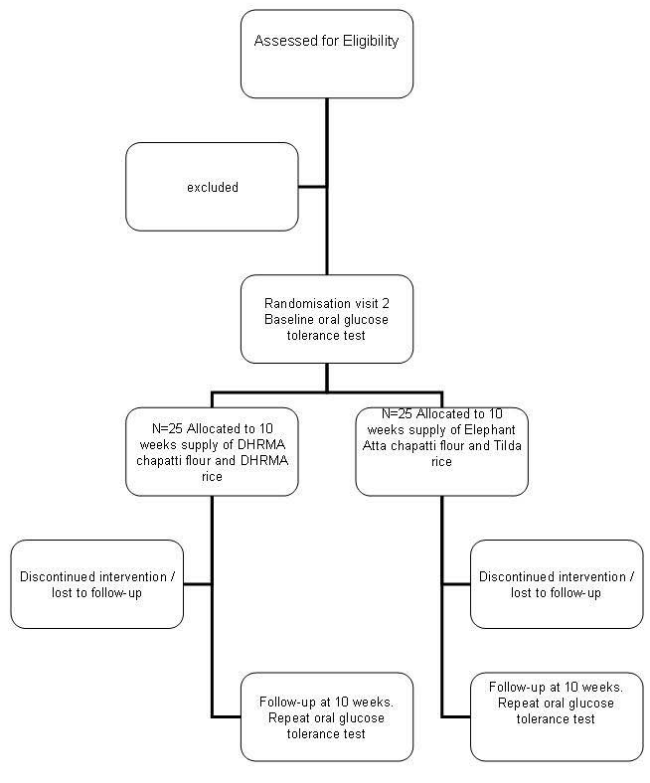
**Study Flow-chart**.

A total of 50 subjects will be randomised onto either arm in a blinded fashion, and supplied with study carbohydrates (see Figure 1). The study carbohydrates will be packaged by our industrial collaborator (East End Foods, UK), who randomise and allocate individual packet numbers to each test food (information relating to the contents will be stored with our collaborator and so blinded to study researchers and the study participants). The supply of rice and flour will be in clear, unlabelled 15 kg bags packaged within a white box with an assigned randomisation number and trial details. Random number allocation will be through a computer generated system, with no provision for factorisation.

Each participant will be asked to attend three visits (screen, randomisation and follow-up). The screening visit will be used to initiate the consent process blood, and to assess inclusion criteria (eg dietary intake).

At randomisation and follow-up, blood pressure and anthropometry will be measured and an OGTT will be performed. Subjects will also be asked to complete a 4-day dietary record at randomisation and follow-up. The main metabolic measures will be 10 week changes in - OGTT metabolites and hormones (glucose, insulin, NEFA), adiponectin, leptin and lipids and lipoproteins (including serum triglycerides and HDL cholesterol).

### Power calculation

The sample size was calculated on the results from our migration study [13] and dietary intervention study [23] where we found a 0.56 (SD = 0.34) fall in CHD risk using the biomarker C-reactive protein for individuals on the substitution dietary intervention and no significant reduction in the other groups. Using a paired t-test, at 80% power, α<0.05, 25 subjects/arm will allow the detection of a 1 mmol difference in 2 hr glucose and 0.15 mmol change in NEFA suppression (this is ½ the difference seen between glucose tolerant and intolerant Indian Gujaratis in our migration study [14]).

## Discussion

The DHRMA study aims to investigate key staples in the South Asian diet and provides the basis for culturally specific dietary options for the management of CHD risk, in addition to a better understanding of the cardiovascular-metabolic risk in this group. The research focuses on the efficacy of low glycaemic index foods, which have been shown to be beneficial in relation to metabolic processes, and specifically, those that are reported to contribute to cardiovascular risk amongst South Asian populations.

The DHRMA study makes the assumption that it is the quality of carbohydrate that influences cardiovascular risk in South Asians. From our previous work we observed that it was a transition from a diet high in carbohydrates to one with reduced energy from carbohydrates that promoted an adverse risk in South Asians [13]. However, we were not able to analyse the quality of the carbohydrate. Some key nutritional deficiencies have been highlighted for this population, which are not under investigation in the current study. For example, South Asians living in Britain also have both lower and altered vitamin D metabolism [24,25], which is consistent with observations amongst migrant South Asians elsewhere [26]. Long chain omega-3 fatty acids (Omega-3 PUFA) are accepted as a treatment for hypertriglyceridaemia and provide protection against CHD [27,28]. Low levels of omega-3 fattys acids have been consistently reported as a risk factor of CHD in South Asians living in urban, rural and UK settings [29-32].

By using foods from the Indian subcontinent, the DHRMA study aims to regenerate traditions in diet, likely to be effective in elderly South Asians, and will also provide an additional management angle for clinicians. Hence, the data will provide an evidence base for a 'tailored approach' to cardiovascular risk management in this high risk population.

## Competing interests

The authors declare that they have no competing interests.

## Authors' contributions

JVP conceived and designed the study, and drafted the manuscript. All authors have participated in the design and co-ordination of this research, the acquisition of funding, and have read and approved the final manuscript.

## Funding

The DHRMA study was funded by the British Heart Foundation (Project Grant Award).

## Pre-publication history

The pre-publication history for this paper can be accessed here:

http://www.biomedcentral.com/1471-2261/11/70/prepub
